# Health facility-based Active Management of the Third Stage of Labor: findings from a national survey in Tanzania

**DOI:** 10.1186/1478-4505-7-6

**Published:** 2009-04-16

**Authors:** Godfrey S Mfinanga, Godfather D Kimaro, Esther Ngadaya, Sirili Massawe, Rugola Mtandu, Elizabeth H Shayo, Amos Kahwa, Ominde Achola, Alice Mutungi, Rod Knight, Deborah Armbruster, David Sintasath, Andrew Kitua, Cynthia Stanton

**Affiliations:** 1 NIMR Muhimbili Medical Research Centre (MMRC), Dar es Salaam, Tanzania; 2 Muhimbili University of Health and Allied Sciences (MUHAS), Dar es Salaam, Tanzania; 3 National Institute for Medical Research (NIMR-HQ), Dar es Salaam, Tanzania; 4 East Central Southern Africa Health Community, Family, and Reproductive Health Programme, Arusha, Tanzania (ECSA); 5 Regional Center for Quality of Health Care, (Reproductive and Neonatal Health) (RCQHC), Jinja, Uganda; 6 The Johns Hopkins Bloomberg School of Public Health, Baltimore, USA

## Abstract

**Background:**

Hemorrhage is the leading cause of obstetric mortality. Studies show that Active Management of Third Stage of Labor (AMTSL) reduces Post Partum Hemorrhage (PPH). This study describes the practice of AMTSL and barriers to its effective use in Tanzania.

**Methods:**

A nationally-representative sample of 251 facility-based vaginal deliveries was observed for the AMTSL practice. Standard Treatment Guidelines (STG), the Essential Drug List and medical and midwifery school curricula were reviewed. Drug availability and storage conditions were reviewed at the central pharmaceutical storage site and pharmacies in the selected facilities. Interviews were conducted with hospital directors, pharmacists and 106 health care providers in 29 hospitals visited. Data were collected between November 10 and December 15, 2005.

**Results:**

Correct practice of AMTSL according to the ICM/FIGO definition was observed in 7% of 251 deliveries. When the definition of AMTSL was relaxed to allow administration of the uterotonic drug within three minutes of fetus delivery, the proportion of AMTSL use increased to 17%. The most significant factor contributing to the low rate of AMTSL use was provision of the uterotonic drug after delivery of the placenta. The study also observed potentially-harmful practices in approximately 1/3 of deliveries. Only 9% out of 106 health care providers made correct statements regarding the all three components of AMTSL. The national formulary recommends ergometrine (0.5 mg/IM) or oxytocin (5 IU/IM) on delivery of the anterior shoulder or immediately after the baby is delivered. Most of facilities had satisfactory stores of drugs and supplies. Uterotonic drugs were stored at room temperature in 28% of the facilities.

**Conclusion:**

The knowledge and practice of AMTSL is very low and STGs are not updated on correct AMTSL practice. The drugs for AMTSL are available and stored at the right conditions in nearly all facilities. All providers used ergometrine for AMTSL instead of oxytocin as recommended by ICM/FIGO. The study also observed harmful practices during delivery. These findings indicate that there is a need for updating the STGs, curricula and training of health providers on AMTSL and monitoring its practice.

## Background

Postpartum hemorrhage (PPH) is one of the world's leading cause of maternal mortality. PPH occurs in over 10% of all births and is associated with case a fatality rate of 1%. Twenty five percent of all maternal deaths are caused by severe hemorrhage. [1,2] Recent information indicates that worldwide the percentage is even higher than previously thought, ranging from 30–39%. [3] Reports from Tanzania show that maternal deaths due to hemorrhage were 23% in year 1988. [4]

Active management of the third stage of labor (AMTSL) is a feasible and inexpensive intervention that can help to save millions of women's lives. The AMTSL involves three main components: The use of a uterotonic agents within one minute following the birth of the baby, delivery of the placenta with Controlled Cord Traction (CCT) and massage of the uterus after delivery of the placenta [5]

This definition is also supported by the World Health Organization. [1,6] This definition differs from the original research protocol in the Bristo trials which includes immediate cord clamping and do not include massage. [7,8] The FIGO/ICM Joint Statement and the WHO report on *the Managing Complications in Pregnancy and Childbirth*, do not include immediate cord clamping. [9] Studies shows that there is now considerable evidence that early cord clumping does not benefit mothers or babies and may even be harmful. [10,11]

Clinical trials in developed countries show that the use of AMTSL, in contrast to physiologic management of the third stage of labor, significantly reduces PPH. [12] A Cochrane review of these trials concludes by recommending AMTSL for all women delivering in a hospital and anticipating the vaginal birth of a single baby. [12]

Based on this body of evidence, ICM and FIGO issued a joint statement in November 2003, stating that every woman should be offered AMTSL "as a means of reducing the incidence of PPH due to uterine atony." The inclusion of AMTSL in the WHO evidence-based manual *Managing Complications in Pregnancy and Childbirth *also attests to the international acceptance of this practice as the standard of care. Evidence regarding adoption of this practice, however, is limited. Evaluations of donor funded projects incorporating AMTSL tend to be limited to reporting on the numbers of providers trained and the percent achieving competence following training. Apart from anecdotal information, a 2003 article by the Global Network for Perinatal and Reproductive Health offers a limited glimpse into the adoption of this practice [13]. Their results, based on an evaluation of the15 university-based referral obstetric centers in developed and developing countries, show substantial variation between and within hospitals. There is insufficient evidence for drawing conclusions about the effectiveness of this practice in its altered states. These results do suggest, however, that proportions of the service providers using AMTSL is quite low and, where it is practiced, the definition varies within and between countries.

Since 1987, the Safe Motherhood Initiative has stated that maternal mortality is an issue of health infrastructure. AMTSL is a highly measurable, evidence-based, and life-saving aspect of the health infrastructure. Given that PPH is a leading cause of maternal death in many countries with high maternal mortality, there is an important and urgent need for information from these countries on current practices regarding AMTSL. The aim of the study was to provide descriptive information necessary to assess AMTSL practices and identify major barriers to its use. The study was conducted in 15 out of 21 regions of the Tanzania Mainland.

## Methods

### Sample design, size, and study areas

This descriptive study was conducted from November to December, 2005 to provide information on the use of AMTSL and major barriers to its use at national and facility levels. Five types of data were collected to address the study objectives. The data collected include; observations of deliveries, short interviews with key informants regarding procurement of AMTSL drugs and the content of pre- and in-service medical and midwifery education, document review of both the STG and pre-service curricula and teaching materials for midwives and physicians regarding AMTSL, and verification of the availability and storage conditions of AMTSL drugs. The reviewed documents included: 1) Advanced Life Saving Skills, volume 2: module 1–10; Trainees manual-Reproductive and Child Health section, MoH, Tanzania; 2) Proposal for upgrading the diploma in nursing to a University of Dar es Salaam (UDSM), Muhimbili University College of Health Siences (MUCHS), February 2003; 3) Focused antenatal care including prevention and treatment of malaria and syphilis during pregnancy. Reference manual for nurses and midwives. Tanzania, MoH, May 2004; 4) UDSM, MUCHS, Eight semester Curriculum for the BSc Nusing program, September 2002 and 5) UDSM Faculty of Nursing – Proposal for Conversion program, targeted group Advanced diploma in Midwifery Degree, January, 2003

A nationally representative sample of facility-based deliveries was selected. The study team selected the sample using a two-stage design. To begin, 15 of the 21 regions in the Tanzania mainland were selected with equal probability.

These regions included Mtwara, Lindi, Ruvuma, Mbeya, Iringa, Morogoro, Dodoma, Coast, Tanga, Kilimanjaro, Arusha, Manyara, Mwanza, Shinyanga, and Dar es Salaam. Data collectors then met with the regional medical officer (RMO) and regional Maternal and Child Health (MCH) coordinator and obtained a current list of all hospitals with at least two deliveries per day. From this list, researchers randomly selected two hospitals to provide a total of 30 hospitals. However, it was not possible to collect data from one hospital.

The distribution among the remaining 29 hospitals surveyed was as follows: 10 regional hospitals (one was both a regional and referral hospital), 15 district hospitals, and 4 faith-based (church-affiliated) hospitals. Once at a selected hospital, a data collector observed all deliveries over two days for a period of 16 hours per day (7 am to 11 pm). The target sample size was 180 deliveries.

### Training for data collectors

Twelve data collectors were trained to participate in the fieldwork for this study, all of whom were either research scientists or Medical Doctors. The 3-day training consisted of discussion and presentations about various survey questionnaires, role-playing exercise, demonstrations of AMTSL with anatomical models and a pretest at two district hospitals, during which all data collectors had the opportunity to observe at least 9 deliveries under the supervision of a study coordinator.

### Definition of Terms

AMTSL definition: The study used two definitions of AMTSL: Definition A is the FIGO/ICM definition, which involves administration of 10 IU of oxytocin within 1 minute following the delivery of the fetus, CCT, and immediate uterine massage following delivery of the placenta. In cases where oxytocin is not available 0.5 mg of ergometrine IM is recommended. Definition B follows the same criteria as Definition A but relaxes the time requirement for oxytocin administration from 1 to 3 minutes. This is also referred as "adequate" use of the uterotonic drug.

Controlled Cord Traction (CCT): Researchers defined CCT as the application of gentle traction of the umbilical cord, with upward, manual support of the uterus, as a means of delivering the placenta.

### Data management and analysis

Data were double entered and validated using Epi Info™ (Version 6). Following the data cleaning process, descriptive analyses were carried out using STATA (Version 8.0) (College Station, TX, USA). Various proportions for delivery characteristics, providers' knowledge on AMTSL, and AMTSL use according to definitions given were calculated

To reduce bias in the results, researchers applied weights during analysis for both observation of deliveries and provider interviews. Delivery weights corrected for the number of observed deliveries not being in proportion to the number of reported deliveries per year. If, during the observation period, the number of deliveries per day in a facility is less than the average number of deliveries per day for the entire year, the weight will adjust the value to match the number of deliveries per day for the entire year. Conversely, if the number of deliveries during the observation period was greater than the average number of deliveries per day for the entire year, the value was adjusted downward while provider weights adjusted for the number of providers interviewed in relation to all providers managing deliveries in the facility.

If the number of providers interviewed in a facility was less than the number that managed deliveries, the weight adjusts the value to match the number that managed deliveries. If the number interviewed matches the number that manage deliveries, the weight would initially be 1.0, because no adjustment is required. In a few cases, the value was adjusted downward because health practitioners not managing deliveries directly were included in the sample.

The final weights in these cases could differ from 1.0, because another adjustment was made to ensure the overall weighted and unweighted sample sizes match. The n values in all tables represent the weighted values. The results report the weighted sample size, although the overall weighted sample size was designed to match the overall unweighted sample size, as mentioned above.

### Ethical Approval

The protocol for this study was reviewed and approved by the National Ethical Committee in Tanzania and also by the Committee for Human Research at the Johns Hopkins Bloomberg School of Public Health in Baltimore, Maryland USA, which considered it exempt from human subjects research. Parturient women were asked their consent to be observed upon admission to the hospital, or when that was not possible, in the labor and delivery ward. Likewise, providers were asked for their consent to participate in the face to face interviews. No identifiers of any kind were collected.

## Results

### Individual health facility questionnaire

Researchers completed a questionnaire for each health facility (n = 29) in which deliveries were observed. Interviews conducted with facility staff documented the number of deliveries, policy information, and availability and storage conditions of uterotonic drugs in the pharmacy.

### Health care providers

Interviews with health care providers at the maternity ward documented attitudes and perceptions regarding the routine use of AMTSL in those facilities. The study team interviewed a total of 106 providers over a 2 day period in each facility. The providers interviewed included nurse midwives (64.4%), nurse officers (11.3) and others (24.3%). The others category includes Assistant Medical Officers, Mother and Child Health AID, medical attendants, nurse assistants, and student nurses. Many of the interviewed providers were also observed during delivery. With regard to the health care providers' AMTSL knowledge, only 9% made correct statements regarding all three components as in the definition of AMTSL. These included uterotonic drug administered within 1 minute following the delivery of the fetus, CCT and uterine massage. Of all responses, 36% and 46% of providers mentioned one and two components, respectively. In general, 91% of providers made no correct statements regarding the definition of AMTSL.

### Policy, logistics, and drug availability

#### National standard treatment guidelines

The Tanzania Standard Treatment Guidelines (STG) and the 1997 Essential Drug List (EDL) list registered two types of uterotonic drugs: oxytocin and ergometrine. The list indicates that oxytocin (5 IU) should be administered intravenously (IV) for induction and augmentation of labor and intramuscularly (IM) for stimulation of uterus after delivery of fetus. The dose of ergometrine recommended by STG and EDL for prevention of PPH is 0.5 mg/IM.

AMTSL is minimally mentioned in the revised (l997) STG, and the practice undefined. Oxytocin, unlike ergometrine, is limited to hospital use and when prescribed by a physician.

The pre-service curricula and teaching materials for medical doctors and nurse-midwife students as shown in reference material listed in methodology section, do not specifically mention AMTSL. However, the curricula on preventing PPH recommends resources that advocate the use of oxytocin (5 IU/IM) or ergometrine (0.5 mg/IM) after delivery of the baby's anterior shoulder, followed by umbilical cord clamping immediately after delivery and removing the placenta by controlled cord traction. Uterine massage following delivery of placenta is also mentioned in the curricula for the treatment of PPH.

#### Availability of uterotonic drugs

Oxytocin and ergometrine ampoules of 5 IU/ML and 0.5 mg/ML respectively were available at the central pharmaceutical storage site. Misoprostol and Syntometrine^® ^was not in the hospitals and in the government central Medical Store Department (MSD).

Both drugs are stored at a temperature of 2°C to 8°C and restricted from light at the national storage site, where the drugs are also stored in mobile cold boxes during procurement. Several factors determine the quantity of drugs to be procured: the previous monthly consumption rate, purchasing power, and storage and distribution capacity.

Both oxytocin and ergometrine are readily available at health facilities in Tanzania. For example, 97 percent of observed deliveries occurred in facilities with either oxytocin, ergometrine, or both available in the labor and delivery ward.

#### Storage conditions

In nearly three-quarters (72%) of the health facility pharmacies assessed, the storage conditions for oxytocin was between 8°C and 25°C (without freezing). For ergometrine, the storage temperature in 69% of visited facilities was between 2°C and 8°C. About one third (28%) of health facilities stored oxytocin and ergometrine at room temperature.

#### Facility supplies of uterotonic drugs

We found a generally acceptable amount of oxytocin and ergometrine in the facility pharmacies, with most health facilities having a one-month uterotonic drug supply. Drug availability was found to be problematic in certain zones. For example, the Southern Highland zone – with averages 400 deliveries per month – had less than one week's supply of oxytocin. Similarly, the Eastern zone reported enough ergometrine supplies for less than one month, while nearly 750 deliveries were expected in the next month.

#### Use of AMTSL

A total of 251 facility-based vaginal, non-instrumental deliveries were observed in this study. The characteristics of these observed deliveries are shown in Table 1. Almost 90% of the observations were conducted in regional or district hospitals; 6% were in a central referral hospital.

**Table 1:** Distribution of deliveries by facility and characteristics of the mother

Delivery characteristic	%	n	Delivery characteristic	%	N
Type of facility			Qualification of provider		

Central referral hospital	5.7	14.1	Clinical Officers	1.1	2.8
Regional hospital	38.9	96.7	Midwife	71.3	176.8
District hospital	51.0	126.7	Nurse	11.1	2.5
Health center	0.5	1.3	Other*	11.6	28.8
Other	3.9	9.8	Missing	5.1	12.7

Volume of deliveries per year			Age of mother		

<1,200	4.4	10.9	<20 years	17.4	43.3
1,200 – 2,999	14.2	35.4	20–34 years	75.3	187.2
3,000 – 5,499	18.8	46.8	35+ years	7.2	18.0
			
5,500 – 6,999	17.7	43.9	Gravidity		
			
7,000 – 9,999	18.1	45.1	1	32.8	81.5
10,000+	26.7	66.4	2–5	58.0	144.2

Zone			> 5	9.2	22.8

Central	7.0	17.3	Total	100.0	248.6
Eastern	35.1	87.3			
Lake	12.7	31.5			
Northern	16.8	41.8			
Southern	10.4	25.9			
Southern Highlands	14.1	35.1			
Western	3.9	9.7			
			
Total	100.0	248.6			

*Assistant Medical Officers, Mother and Child Health AID, medical attendants, nurse assistants, and student nurses.

Deliveries were observed in seven geographic zones located throughout the Tanzania mainland. The volume of deliveries in selected facilities varied substantially, ranging from low-volume facilities (managing <1,200 deliveries per year; 4%) to high-volume facilities (> 10,000 deliveries per year; 27 percent).

Physicians were not observed in this study because they tend to manage instrumental or more complicated deliveries, which were excluded from this study sample. Consequently, 71% of the observed deliveries were managed by midwives and 11% by nurses. The mean age of mothers was 25.1 years (range: 14 to 46 years). A majority of the mothers were in the 2 to 5 gravidity group (58%) (See table 1).

#### Use of uterotonic drugs

In the sample of observed deliveries, almost all women received an uterotonic drug (ergometrine or oxytocin) at some point during their deliveries (97%). Eight percent of women were induced, and among spontaneous deliveries, 10% were augmented. About two-thirds (64%) of the women observed received ergometrine, a quarter oxytocin and 3% received both. Combination drugs such as Syntometrine^® ^or prostaglandins such as Misoprostol were not used at all. The percent of deliveries for which ergometrine and oxytocin were used was 67 and 31% respectively, with no restrictions on timing, mode of administration or dose.

Table 2 summarizes the use of uterotonic drugs by facility and characteristics of the mothers. Use of ergometrine or oxytocin varied little across these characteristics. Women delivering at facilities managing 7,000 or more deliveries per year were more likely to receive oxytocin compared to women delivering at lower volume facilities. Use of ergometrine was slightly higher among older and high parity women (84 and 78% respectively).

**Table 2:** Distribution of the use of uterotonic drugs during labor, delivery, and the immediate postpartum period

	Oxytocin only (%)	Ergometrine only (%)	Both (%)	Neither (%)	Missing data	Total (%)	N
Total	25.8	63.8	3.0	2.1	5.2	100.0	248.6

Age of mother							

<20 years	20.4	66.8	4.3	0.0	8.6	100.0	43.3
20–34 years	28.3	61.2	2.7	2.8	4.9	100.0	187.2
35+ years	12.6	84.5	2.9	0.0	0.0	100.0	18.0

Gravidity							

1	24.0	66.5	6.7	0.0	2.8	100.0	81.5
2–5	27.5	60.0	1.4	3.7	7.4	100.0	144.2
> 5	21.6	78.4	0.0	0.0	0.0	100.0	22.8

Type of facility							

Referral hospital	11.1	81.5	3.7	3.7	0.0	100.0	14.1
Regional hospital	23.5	65.3	2.0	0.0	9.2	100.0	96.7
District hospital	31.0	59.0	3.1	3.8	3.2	100.0	126.7
Health center	0.0	100.0	0.0	0.0	0.0	100.0	1.3
Other	6.3	81.5	12.1	0.0	0.0	100.0	9.8

Volume of deliveries per year							

<1,200	5.7	83.5	10.8	0.0	0.0	100.0	10.9
1,200 – 2,999	4.1	88.2	5.7	0.0	2.0	100.0	35.4
3,000 – 5,499	13.1	81.2	2.5	0.0	3.3	100.0	46.8
5,500 – 6,999	13.2	71.4	1.2	1.2	13.0	100.0	43.9
7,000 – 9,999	52.1	38.9	4.1	0.0	4.9	100.0	45.1
10,000+	40.1	47.4	1.1	7.2	4.2	100.0	66.4

In-service training in selected facility:							

For midwives	19.1	73.6	2.6	0.5	4.3	100.0	96.3
For nurses	19.1	73.6	2.6	0.5	4.3	100.0	96.3
For doctors	10.5	78.8	6.2	1.3	3.3	100.0	39.8

Among women receiving ergometrine, which in Tanzania appears to be the drug of choice during delivery, 3% of women received this drug during delivery of fetus, 42% of women received this drug after delivery of the fetus, and 45% received it after delivery of the placenta. An additional 10% of cases had ergometrine administered during delivery of the placenta. Among women receiving oxytocin, about 12% received before delivery of fetus one third (35%) received it following delivery of the fetus, 6% received it during delivery of the placenta, and nearly half (47%) received it following delivery of the placenta.

## Use of AMTSL

Overall, only 7% of deliveries met the criteria for correct use of AMTSL when ergometrine is used. This percentage increases to 17 when the restriction regarding timing of the administration of ergometrine is relaxed to within three minutes of the delivery of the fetus. The percentage of AMTSL use by Definitions A and B vary similarly by characteristics of the mother and facility. In general, women under 20 and over 35 years of age are more likely to have had AMTSL, as are low and high parity women respectively. The largest differences in the use of AMTSL are by geographical zone. In the Central zone, 23% and 40% of deliveries met the criteria for Definitions A and B, respectively. In the Lake and Northern zones, however, none of the deliveries met the criteria for Definition A but 6% and 13% of observed deliveries met the criteria for Definition B.

### Elements of AMTSL

#### Controlled Cord Traction (CCT), and uterine massage

Observers could not detect if this action was taken only after detecting signs that the placenta was beginning to separate from the uterine wall (as specified in the FIGO/ICM recommendation). According to the study's definition, providers performed CCT in over two-thirds (69%) of observed deliveries. Nearly 88% of all deliveries benefited from immediate uterine massage following delivery of the placenta. The study did not document palpation of the uterus at 15 minute intervals following delivery of the placenta.

Cord clamping in within one minute of fetal delivery was practiced in three quarters of facility-based deliveries in Tanzania. Researchers observed cord clamping within two to three minutes of delivery with a large majority of the remaining deliveries

The restrictions included in the definition of adequate relative to overall use, cause a drop of 44 percentage points among births with ergometrine (dropping from 67 to 23%). The use of ergometrine during or following delivery of the placenta accounts for virtually all of this decrease. None of the deliveries met the criteria for adequate use when oxytocin is used. The decrease from 31% to 0 is due to both the administration of oxytocin at times other than following the delivery of the fetus, and to the other dose than the one recommended by ICM/FIGO. In 83% of the deliveries where oxytocin was used, 5 versus 10 IU of oxytocin were administered. The correct use of uterotonic drugs is the same definition as adequate use, with the further restriction that the uterotonic drug must be administered within one minute of the delivery of the fetus. With the restriction of administered uterotonic drug within one minute of the fetus delivery, further decreases AMTSL use from 23% to 9% among deliveries received ergometrine. The additional requirements of controlled cord traction and uterine massage among the providers using ergometrine, further reduce correct use of AMTSL from 9 to 7%.

#### Potentially harmful practices

This study identified four potentially harmful practices in 251 observed deliveries. These practices include the application of fundal pressure while awaiting the placenta (44%), uterine massage following delivery of the fetus (34%), and application of cord traction without manual support of the uterus (21%). All of these practices can increase the risk of PPH or cause problems such as uterine inversion.

## Discussion

The findings of low correct use of AMTSL using ergometrine and non using oxytocin, reflect weakness in the country STG which includes only two components: the drug used (ergometrine) and CCT. The guidelines fail to mention uterine massage following delivery of the placenta.

The correct use of AMTSL according to ICM/FIGO definition, does prevent PPH by 60% and it is established that about 1/3 of the MMR is due to PPH. [1-4] However, the low rate of correct use of AMTSL practice, use of ergometrine and suboptimal dosages of oxytocin are major barriers to effective PPH prevention in the country and this could slow down achievement of the MDGs of reducing the MMR by 75% by 2015. [14] The low use of correct AMTSL which is reported in this study has also been observed elsewhere in the developed and developing countries. [13]

Two practices are primarily responsible for low compliance with the official definition of AMTSL: using uterotonic drugs during or following delivery of the placenta (versus immediately following the delivery of the fetus), and using uterotonic drugs within three (versus one minute) following delivery of the fetus. The wrong timing of administering uterotonic drugs was observed in about half of deliveries in which ergometrine or oxytocin was either administered before delivery of the fetus or after delivery of placenta. This is in contrast to the FIGO/ICM definition, recommending use of a uterotonic within one minute of the delivery of the fetus. [5,15]

In addition, the observed poor AMTSL knowledge among the providers interviewed reflect weakness in training programs. The observed poor knowledge is in sharp contrast to the 93% who claimed they received AMTSL training either during pre-service education, or with in-service training.

It is, therefore, important for the Ministry of Health and Social Welfare (MOHSW) to facilitate a process of updating national STG guidelines to include using of the recommended dose of oxytocin (10 IU) and all AMTSL components including proper massage, and the appropriate timing of administration of a uterotonic drug (following the delivery of the fetus) as recommended for AMTSL by ICM/FIGO. [5,15]

The concerned authorities for learning institutions should facilitate the process of incorporating the correct use of AMTSL according to ICM/FIGO definition into both pre-service and in-service training materials, and provide refresher courses for MOHSW staff managing deliveries in the country.

This study finding of a number of potentially harmful practices including applying fundal pressure while waiting for delivery of the placenta, gentle traction on the cord without external support of the uterus, and external massage of the uterus while waiting for delivery of the placenta, raise another concern in PPH control and maternal health. All of these practices increase the possibility of PPH or other problems such as uterine inversion. Pre-service and in-service training should specifically emphasize the potential danger to women when these practices are used.

The U.S Pharmacopeia has changed their guidance on storage of oxytocin from 15°C to 25°C to a narrower range of 2°C to 8°C in the last few years. A recent review of this change questioned the stringency of this requirement. It should also be noted that research has identified that oxytocin can remain at room temperature up to 30°C for 3 months. [16] In this study most of facilities stored oxytocin and ergometrine properly. However, about one third of the facilities improperly stored ergometrine at room temperature. Most of the health facilities were found to have uterotonic drug supplies enough for the next one month only. Since oxytocin can be stored at room temperature for up to three months[16], the facilities could have ordered oxytocin stock enough for the next three months instead of one month. However, storage at room temperature should not be encouraged since we can not ensure that such storage will not exceed three months.

The routine use of AMTSL, as recommended, is likely to increase the use of oxytocin in the country. The procurement, distribution and storage policies should be reviewed to ensure that sufficient oxytocin supplies are available and properly stored in hospitals, health centers, and clinics. The policies should also ensure that there is increased use of oxytocin and decreased ergometrine use to comply with WHO, FIGO, and ICM standards. In addition, oxytocin is effective in 2–3 minutes after injection, has minimal side effects, could be used in all women and is more stable in storage than ergometrine. [16-18]

It is important to monitor and evaluate the use of AMTSL using the updated definition, since AMTSL shows to reduce the incidence of PPH, shortening of the third stage of labor and reducing the need for additional treatments. [9,17,18] Therefore, we recommend that supervisors are trained in AMTSL and include items on the supervision checklists to ensure its use as an indicator of quality. Labor and delivery logbooks should include space to note and monitor AMTSL use. Either the practice of implementing clinical audits on AMTSL should be established.

The study was designed to assure a nationally representative sample of vaginal facility-based births. However, the sample was restricted to public facilities due to difficulties to obtaining permission to observe deliveries in private facilities and limited funds. However, the four faith based hospitals were included because they also served as designated district hospitals. Even though, the private hospitals offering obstetric services are few and most them are located in major cities, the four faith based hospitals are not enough to fully describe AMTSL practice in private hospitals.

In addition, the study sample was restricted to deliveries occurring at national referral, regional and district hospitals so that we could be sure of observing at least one delivery per day. It was also of interest to compare AMTSL practice in both low and high level facilities. Furthermore, the sample size is not adequate for regions, districts or facility based comparative analysis and therefore the interpretation of our finding is limited at national level.

## Conclusion

Correct practice of AMTSL according to the ICM/FIGO definition was observed in 7% using ergometrine and non using oxytocin. All providers used ergometrine for AMTSL instead of oxytocin as recommended by ICM/FIGO. The study observed harmful practices which included the application of fundal pressure while awaiting the placenta (44%), uterine massage following delivery of the fetus (34%), and application of cord traction without manual support of the uterus (21%). Only 9% of providers responded correctly as regarding all three components as in the definition of AMTSL. The drugs for AMTSL were available in nearly all health facilities and were stored at the right conditions in over 70% of the facilities visited. The content of the training curricula for midwives and physicians does include AMTSL as recommended by FIGO/ICM. The study also observed harmful practices during delivery. These findings indicate that there is need for updating the STGs, curricula and training of health providers on AMTSL and monitoring its practice.

## Competing interests

The authors declare that they have no competing interests.

## Authors' contributions

SGM, SM, RK, OA, DS, DA, CS, PB, and JT participated in the design of the study and offered technical support. DGK, EN, RM, ES and AK participated in data collection and management, and manuscript writing.
